# Rice *TSV2* encoding threonyl-tRNA synthetase is needed for early chloroplast development and seedling growth under cold stress

**DOI:** 10.1093/g3journal/jkab196

**Published:** 2021-07-30

**Authors:** Dongzhi Lin, Wenhao Zhou, Yulu Wang, Jia Sun, Xiaobiao Pan, Yanjun Dong

**Affiliations:** 1 College of Life Sciences, Shanghai Normal University, Shanghai 200234, China; 2 Crop Institute, Taizhou Academy of Agricultural Sciences, Zhejiang Linhai 317000, China; 3 Shanghai Key Laboratory of Plant Molecular Sciences, Shanghai 200234, China; 4 Institute of Genetics, Shanghai Normal University, Shanghai 200234, China

**Keywords:** albino phenotype, chloroplast development, cold stress, rice, threonyl-tRNA synthetase

## Abstract

Threonyl-tRNA synthetase (ThrRS), one of the aminoacyl-tRNA synthetases (AARSs), plays a crucial role in protein synthesis. However, the AARS functions on rice chloroplast development and growth were not fully appraised. In this study, a thermo-sensitive virescent mutant *tsv2*, which showed albino phenotype and lethal after the 4-leaf stage at 20°C but recovered to normal when the temperatures rose, was identified and characterized. Map-based cloning and complementation tests showed that *TSV2* encoded a chloroplast-located ThrRS protein in rice. The Lys-to-Arg mutation in the anticodon-binding domain hampered chloroplast development under cold stress, while the loss of function of the ThrRS core domain in TSV2 fatally led to seedling death regardless of growing temperatures. In addition, *TSV2* had a specific expression in early leaves. Its disruption obviously resulted in the downregulation of certain genes associated with chlorophyll biosynthesis, photosynthesis, and chloroplast development at cold conditions. Our observations revealed that rice nuclear-encoded *TSV2* plays an important role in chloroplast development at the early leaf stage under cold stress.

## Introduction

Aminoacyl-tRNA synthetase (AARS) is an important component of protein synthesis in ribosomes and is essential for translation in three different compartments of the plant cell: chloroplasts, mitochondria, and the cytosol (Berg *et al.* 2005; Sang *et al.* 2005). AARSs generally consist of catalytic core domain, binding zinc ion domain, insertion domain, anticodon-binding domain, and editing domain (Greenberg *et al.* 2008) and involves in the process of amino acid transfer to its homologous tRNA (O'Donoghue and Luthey-Schulten 2003). AARS and tRNA play a key role in the first step of protein synthesis. According to the homology and folding pattern, AARSs can be grouped into Class I and Class II, and each AARS class can be further divided into three subclasses: A, B, and C (Martinis *et al.* 1999).

Previous studies have shown that AARS not only plays a critical role in protein synthesis but also participates in and regulates various biological processes, including RNA transcription and splicing, protein translation, signal transduction, and cell apoptosis (Hausmann and Ibba 2008). In *Arabidopsis thaliana*, several mutants involving in AARS genes have been identified. In the *edd1* mutant, inactivation of *GLYRS* encoding glycyl-tRNA synthetase causes embryonic development to stagnate, ultimately leading to the death (Uwer *et al.* 1998). Mutations in the *PRORS1* encoding prolyl-tRNA synthetase in *Arabidopsis* thaliana lead to abnormal transcription levels of the photosynthesis and plastid-synthesis genes, also leading to seedling death (Pesaresi 2006). The mutations of *NbERS* and *NBRS*, encoding glutamyl-tRNA synthetase and serine-tRNA synthetase, respectively, resulted in abnormal chloroplast and reduction of chlorophyll content, and finally etiolated leaf phenotype (Kim 2005). As for ThrAS in *A. thaliana*, though the phenotype (albinism and embryo death) observation of the mutant and specific expression localization has been carried out, its molecular mechanism is, however, still unclear (Berg *et al.* 2005; Duchêne *et al.* 2005).

Rice is the most important food crop in Asia and has been set up as a model species for genome study. Low temperature is a serious abiotic stress in rice production, which hinders a broad spectrum of cellular components (e.g. chloroplast), metabolisms (e.g. photosynthesis), plant growth, and yield. In spite of the facts that some indispensable genes for chloroplast development/seedling growth in rice at low temperatures have been identified, such as *TCD9* (Jiang *et al.* 2014), *V1* (Kusumi *et al.* 2010b), *V2* (Sugimoto *et al.* 2007), *V3* (Yoo *et al.* 2009), *OsV4* (Gong *et al.* 2014), *TCD5* (Wang *et al.* 2016a), *TCD10* (Wu *et al.* 2016), *TCD11* (Wang *et al.* 2017), *TSV3* (Lin *et al.* 2018), *TCM12* (Lin *et al.* 2019), *TCD33* (Wang *et al.* 2020), and *TCD3* (Lin *et al.* 2020), the molecular mechanism of cold resistance and its impact factors are not well established (Wang *et al.* 2020). In addition, studies involving mutants caused by AARS mutations were rarely reported in rice, except for *OsValRS2* encoding Val-tRNA synthetase and its mutation leading to damage of chloroplast development and chloroplast ribosome biogenesis (Wang *et al.* 2016b), *LAS* encoding ThrAS with its mutation leading to albino-lethal seedling, regardless of growing temperatures (Zhang *et al.* 2017), and *OsERS1* encoding glutamyl-tRNA synthetase and its mutation inducing male-sterility (Yang *et al.* 2018). To our knowledge, the thermo-sensitive lethal mutants involving in AARS genes have not been reported in rice yet to date. In this study, the *tsv2*, a thermo-sensitive lethal mutant of threonyl-tRNA synthetase (ThrRS) gene that exhibits albino-lethal seedling under cold stress but not under normal conditions, was identified and characterized. Apparently, the chloroplast-localized *TSV2* plays a vital role in chloroplast development and seedling growth under cold stress in rice.

## Materials and methods

### Plant materials and growth conditions

The rice *thermo-sensitive virescent mutant tsv2* was discovered in our mutant pool from Jiahua 1 [wild type (WT), *japonica* variety] treated with Cobalt-60 gamma rays. The mutant phenotype was distinguishable from normal green at Hainan Island, China (winter season, subtropical climate) and Shanghai, China (spring season, temperate climate) under local conditions during the early seeding stage. The *tsv2* mutant was crossed with Peiai64S (*indica* variety) and the obtained F_2_ seeds were used for genetic analysis and gene cloning. WT and *tsv2* plants were cultured in incubators under controlled 12 h of light and 12 h of dark at a constant temperature of 20, 24, 28, and 32°C, respectively, for phenotypic characterization, photosynthetic pigment analysis, and DNA and RNA extractions.

### Measurement of photosynthetic pigments and transmission electron microscopy

For the photosynthetic pigment analysis, 200 mg of fresh leaves were taken from the 3-leaf-stage seedlings cultured at 20, 24, 28, and 32°C and incubated with 5 mL of extraction buffer (ethanol:acetone:water = 5:4:1) at 4°C in the dark for 18 h. Using spectrophotometer (Beckman Coulter, Danvers, MA, USA), chlorophyll *a*, *b*, and Car contents were measured according to the modified methods of Arnon (1949) and Wellburn (1994). This experiment was carried out in three biological replicates.

To observe ultrastructure of chloroplast, tissues from the 3rd leaves of the 3-leaf-stage WT and *tsv2* seedlings, grown at 20 and 32°C, respectively, were sampled and treated with the mixed solution of 3% glutaraldehyde solution and 2.5% paraformaldehyde. The observation of chloroplast was performed following Jiang *et al.* (2014). Samples were viewed under a Hitachi765 (Hitachi, Tokyo) transmission electron microscope (TEM).

### Mapping and cloning of *TSV2* gene

Total genomic DNA from rice fresh leaves were extracted by CTAB method described in Murray and Thompson (1980). DNA-specific fragments were amplified by EDC-810 PCR instrument (Eastwin, Shanghai, China). PCR products were transferred to 2–4% agarose gel containing ethidium bromide for electrophoresis. The bands were observed and recorded under UVP imager. The F_2_ population of 1308 individuals with the mutant phenotype was used for fine mapping of the *TSV2* locus. First of all, ninety-two SSR primers based on the Gramene database (http://www.gramene.org) were used to investigate the chromosome of the target gene, and then developed SSR and InDel markers (Supplementary Table S1) were used for fine-mapping of *TSV2*. Next, DNA fragments of the candidate genes were PCR amplified and sequenced (SinoGenoMax, Shanghai, China). Lastly, the function and open reading frames of the candidate genes were obtained from TIGR (http://rice.plantbiology.msu.edu/cgi-bin/gbrowse/rice/) and conserved domain structures were predicted using SMART (http://smart.embl-heidelberg.de/).

### Complementation experiment

First, the WT genomic DNA fragment covering the entire *TSV2* (*LOC_Os02g33500*) coding region (3.9 kb), plus a 1.4-kb upstream region and a 0.6-kb downstream sequence was amplified using the specific primers, pF: 5′-TACGAATTCGAGCTCGGTACCTCCACCAAAGTTTACGAAGC-3′ (*Kpn*I) and pR: 5′-GCTGTGAAGAACCTCCCTATGTCGACCTGCAGGCATGCAAG-3′ (*Sal*I). The underlined sequences stand for cleavage sites of the restriction enzymes. Then, the amplified fragment was cloned onto vector pCAMBIA1301 (CAMBIA, http://www.cambia.org.au), the pCAMBIA1301-TSV2 plasmids were transferred into *Agrobacterium* EHA105 and introduced into the *tsv2* mutant by *Agrobacterium tumefaciens*-mediated transformation (Hiei *et al.* 1994), except that the temperature used for in *vitro* plant differentiation was set at 20°C. The genotype of transgenic plants was determined using PCR amplification of the *hygromycin phosphotransferase* gene (*HPT*) with primers *HPTF* (5′-GGAGCATATACGCCCGGAGT-3′) and *HPTR* (5′-GTTTATCGGCACTTTGCATCG-3′) and GUS gene with primers GUSF (5′-GGGATCCATCGCAGCGTAATG-3′) and GUSR (5′-GCCGACAGCAGCAGTTTCATC-3′) as selection. In addition, all T_1_ seedlings were cultured at 20°C and were used to examine the segregation of the mutant phenotype.

### Targeted mutation of *TSV2* gene

To determine if the novel allelic mutants in *TSV2* display the similar or more severe phenotype compared with the *tsv2* mutant, CRISPR/Cas9 technique was used for the targeted mutation. First of all, adaptor primers (pF1: 5′-GCCGGGCTCAGCTCCGTCTCGTT-3′ and pR1: 5′-AAACAACGAGACGGAGCTGAGCC-3′; pF2: 5′-GTTGGCGGGATCCGACGGCAAGG-3′ and pR2: 5′-AAACCCTTGCCGTCGGATCCCGC-3′) were designed by CRISPR Primer Designer (Naito et al. 2015). The sequence was inserted into the region between the OsU6 promoter and the gRNA scaffolds, from pYLgRNA-OsU6vector, of Cas9 expression backbone vector (pYLCRISPR/Cas9-MH) at the *Bsa*I sites according to the previous methods (Ma *et al.* 2015). The vector was transformed into the WT callus through *A. tumefaciens*-mediated infection (Hiei *et al.* 1994), except that the temperature used for in *vitro* plant differentiation was set at 20°C. The genotype of T_0_ transgenic plants was determined using PCR amplification of the *hygromycin phosphotransferase* gene (*HPT*) with primers *HPTF* (5′-GGAGCATATACGCCCGGAGT-3′) and *HPTR* (5′-GTTTATCGGCACTTTGCATCG-3′). Then, the edited-sites in T_0_ and T_1_ plants were determined by sequencing using PCR amplification of *TSV2* gene with primers *TSV2F* (5′-ACCTCCACCAAAGTTTACGAAGC-3′) and *TSV2R* (5′-CTATGTCGACCTGCAGGCATGCAAG-3′). Lastly, all T_1_ edited seedlings were grown at both 20 and 32°C, respectively, to observe the phenotype and the segregation.

### Subcellular localization of *TSV2*

To determine the subcellular localization of TSV2, the cDNA fragment of *TSV2* gene was amplified by PCR using the corresponding primer pairs (pF: 5′-GAAGATCTATGGCGGCCGCCGTCTCCGC-3′ and pR: 5′-GGGGTACCCCGGTGTGGCGGATGCGGAGCA-3′). The PCR products were cloned into the pMON530-GFP vector, which was transformed into tobacco (*Nicotiana tabacum*) mesophyll cells through Agrobacterium-mediated infection. Meanwhile, empty pMON530-GFP vector was used as control. The analysis was carried out based on previously described method (Jiang *et al.* 2014).

### Sequence and phylogenetic analyses

The protein domain prediction website InterPro was used to analyze the sequence and domain of TSV2, and Phyre2 (http://www.sbg.bio.ic.ac.uk/phyre2/html/) was used to predict the three-dimensional structure of protein. NCBI (http://www.ncbi.nlm.nih.gov) was employed to query the homologous proteins of *TSV2* in other species, and MEGA6 and DNAMAN were used for phylogenetic tree analysis and homologous sequence alignment.

### Transcriptional expression analysis

Total RNA was isolated from fresh tissues (root, stem, leaf, panicle) of WT and *tsv2* plants using an RNA Prep Pure Plant Kit (TianGen, Beijing, China). The transcription of chlorophyll synthesis, chloroplast development, photosynthesis-associated genes (*Cab1R*, *CAO*, *HEMA*, *YGL1*, *LHCP*II, *PORA*, *PsaA*, *PsbA*, *RpoB*, *RpoC*, *Rps20*, *Rps21,16SrRNA*, *23SrRNA*) and temperature-sensitive genes for chloroplast development (*TCD5*, *TCD9*, *TCD10*, *TCD11*) (Supplementary Table S2), in rice was assessed using quantitative real-time PCR (RT-qPCR). The specific primers for qPCR are listed in Supplementary Table S2. A SYBR Premix Ex Taq™ RT-PCR Kit (Takara, Japan) was performed according to manufacturer’s instructions. ABI-7500on Real-Time PCR System (Applied Biosystems; http://www.appliedbiosystems.com) was used to perform the analysis. The relative quantification of gene expression data was analyzed as previously described (Livak and Schmittgen 2001). *Actin* was used as an internal control. This experiment was carried out with four biological replicates.

## Results

### Phenotypic characterization of the *tsv2* mutant

**Figure 1:**
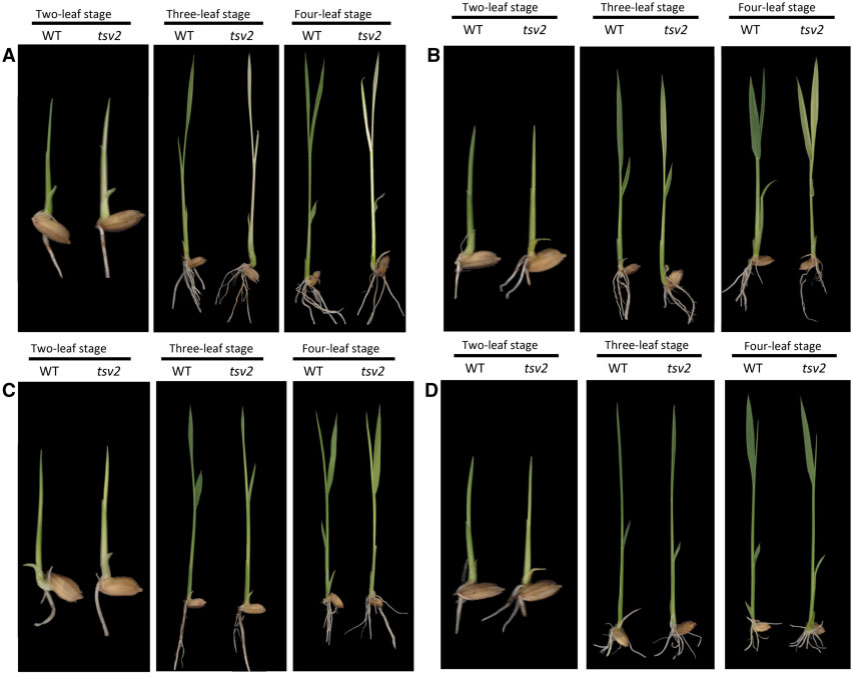
Phenotypic characterization of the *tsv2* mutants. Seedlings of Jiahua1 (WT, left) and *tsv2* mutant (right) at the 2-, 3-, and 4-leaf stages grown at (A) 20°C, (B) 24°C, (C) 28°C, and (D) 32°C.

The growths of *tsv2* and WT seedlings were observed under four growing temperatures (20, 24, 28, and 32°C) (Figure 1). All WT seedlings expectedly displayed green normal phenotype, regardless of temperatures and leaf-stage. However, the *tsv2* mutant was albino phenotype from the beginning and died after 4-leaf stage at 20°C (Figure 1A). At 24°C, the *tsv2* mutant turned to yellow but not lethal (Figure 1B). At 28°C, leaves of *tsv2* were close to green (Figure 1C). Interestingly, at 32°C, the *tsv2* mutant exhibited the normal green as WT plants (Figure 1D). The observations suggest that *tsv2* is a low-temperature sensitive lethal mutant.

**Figure 2:**
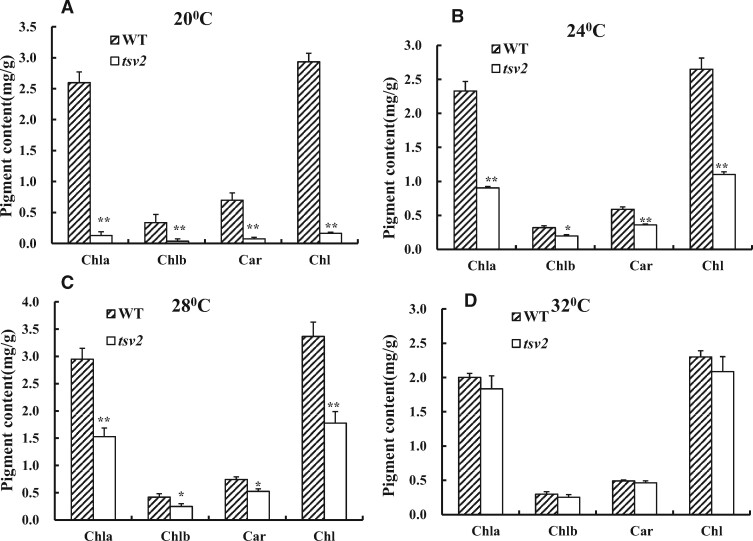
Chlorophyll contents of the 3-leaf stage of WT and *tsv2* seedlings grown at (A) 20°C, (B) 24°C, (C) 28°C, and (D) 32°C, respectively. Data are mean ± SD (*n* = 3). Asterisks indicate statistically significant difference compared with WT by Student’s *t*-test. *P <* *0.05 and **P <* *0.01 are indicated.

**Figure 3:**
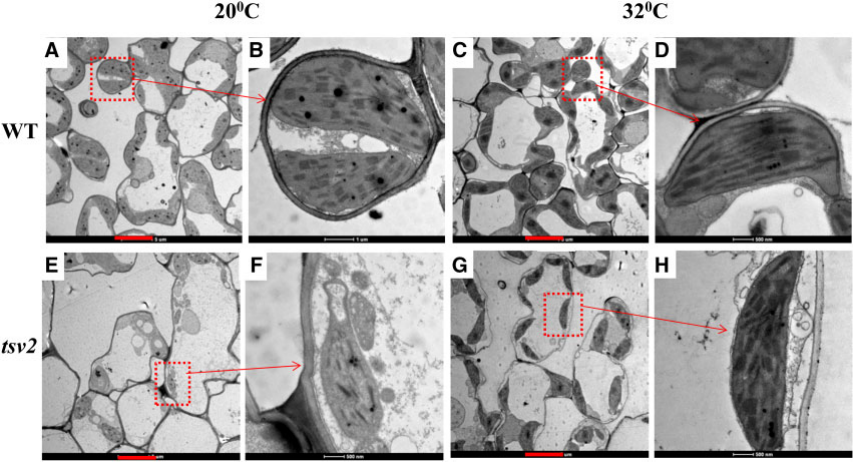
TEM images of the 3rd-leaf chloroplasts in WT and *tsv2* mutants grown at 20°C (A, B, E, F) and 32°C (C, D, G, H). The red scale bar represents 5 μm.

Consistent with the phenotype, the contents of photosynthetic pigments in *tsv2* mutants were the lowest at 20°C (Figure 2A), and gradually upraised to the WT level (Figure 2, B–D) with the increase of temperatures, indicating that chlorophyll accumulation in *tsv2* mutants was suppressed under low temperature. By TEM observation, WT mesophyll cells were found to contain a lot of uniform chloroplasts, irrespective of temperatures (Figure 3, A–D). Nevertheless, the majority of cells in *tsv2* mutants at 20°C contained only few chloroplasts and the structure was abnormal with the grana in the inner capsule decreased obviously (Figure 3, E and F). Interestingly, at 32°C, the *tsv2* chloroplasts were not obviously different from WT plants (Figure 3, G and H). It was then speculated that aberrant chloroplast of *tsv2* resulted in the accumulation alleviation of chlorophyll and mutant phenotype under cold stress.

### Map-based cloning of *TSV2*

**Figure 4:**
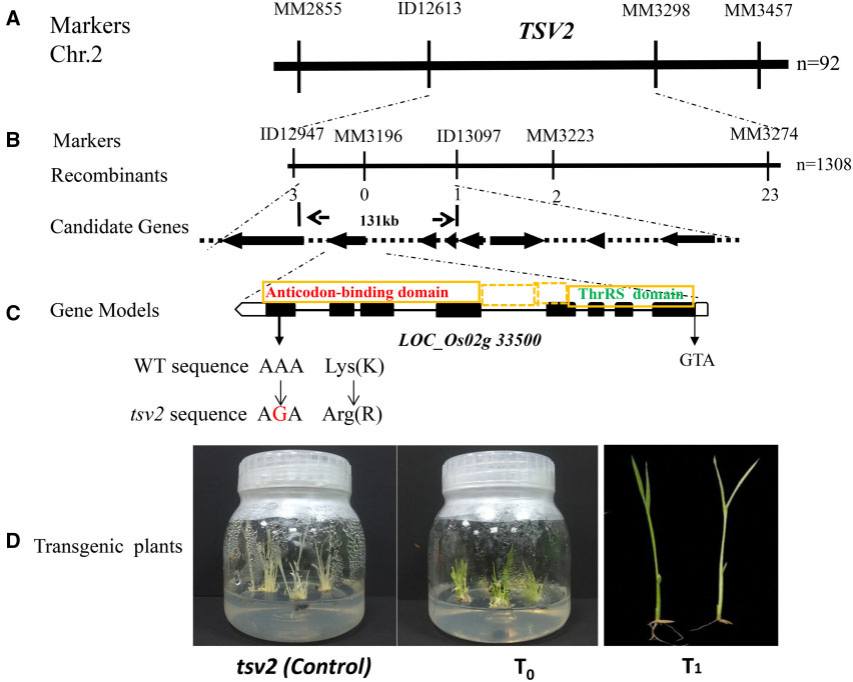
Map-based cloning of the *TSV2* gene. (A) Location of the *TSV2* was localized on chromosome 2. (B) *TSV2* gene was narrowed to 131-kb region, containing eight candidate genes. (**C**) Black boxes represent exons and the black lines between them represent introns. (D) T_0_ transgenic plants transformed with pCAMBIA1301 (control) and pCAMBIA1301-TSV2 at 20°C. The T_1_ segregation from the T_0_ plants at 20°C. The genotypes of green phenotype are *TSV2:TSV2*/*TSV2:tsv2* (left) and the genotype of the albino phenotype is *tsv2:tsv2* (right).

To understand the molecular mechanism underlying the *tsv2* mutant phenotype, map-based cloning was performed to identify the *TSV2* locus. In view of an approximately 3 (green):1 (albino) ratio (χ^2^ = 0.488 < χ^2^_0.05_ = 3.84) in F_2_ segregating population (Supplementary Table S3), consisting of 231 green plants and 70 albino plants at 20°C, this showed the mutant phenotype was controlled by a recessive nuclear gene (*tsv2*). First, ninety-two F_2_ mutant individuals were used for initial mapping, and the target gene *TSV2* was located between ID12613 and MM3298 molecular markers on chromosome 2 (Figure 4A). Subsequently, the mapping F_2_ population was expanded to 1308 individuals, and the *TSV2* gene was narrowed to 131 kb between ID12947 and ID13097, including eight candidate genes (Figure 4B). Sequence and analyze of all candidate genes found that, only a A-to-G mutation in the last exon of *LOC_Os02g33500*, which contains eight exons (http://rice.plantbiology.msu.edu/index.shtml) occurred, resulting in Lys(K)-to-Arg(R) mutation of the 614th site in TSV2 (Figure 4C).

### Complementation of the *tsv2* mutants

To assert that the mutation of *LOC_Os02g33500* was responsible for the *tsv2* phenotype, an expression plasmid containing the entire *TSV2* (*LOC_Os02g33500*) driven by its endogenous promoter was constructed and transformed into the *tsv2* mutants. To speed up, we intentionally induced the differentiation of rice callus at 20°C companying the uninfected calli as a control. Resultantly, 15 T_0_ transgenic seedlings harboring pCAMBIA1301: TSV2 were apparently green as WT plants, while control seedlings remaining albino phenotype (Figure 4D). This acknowledges that *LOC_Os02g33500* can rescue the mutant phenotype. In addition, the segregation of albino phenotype in the transgenic T_1_ population was found at 20°C (Figure 4D). All those together assure that *LOC_Os02g33500* is *TSV2*.

### Characterization of TSV2 protein

Bioinformatic assay revealed that *TSV2* encodes ThrAS protein, consisting of 675 amino acids, with a molecular mass of ∼76.9 kDa, which belongs to Class A in Class II of AARS family. The ThrAS contains at least two domains, including core ThrAS domain and anticodon-binding domain (Figure 4C, Supplementary Figure S1). It was noted that the Lys-to-Arg mutation in *tsv2* mutant occurred in the anticodon-binding domain (Figure 4C, Supplementary Figure S1).

**Figure 5:**
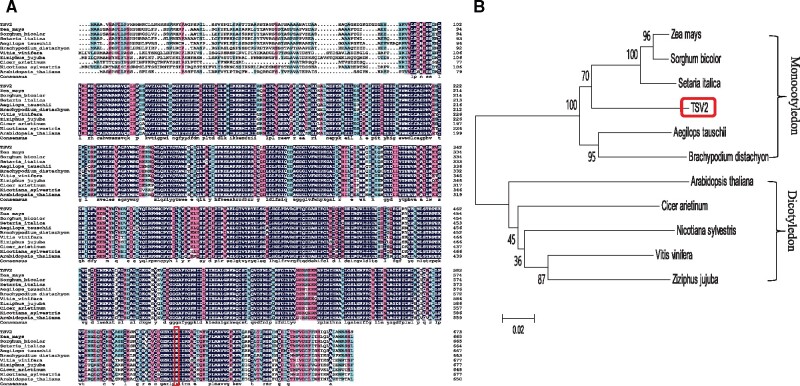
Phylogenic analysis of TSV2 protein. (A) Amino acid sequence alignment of TSV2 with the eleven homologous proteins from amino acids fully or partially conserved are shaded black and gray, respectively. (B) Phylogenic tree of TSV2 and homologous proteins. Scale represents percentage substitution per site. Statistical support for the nodes is indicated.

Orthologs of rice TSV2 were identified in *Zea mays*, *Sorghum bicolor*, *Setaria italic*, *Aegilops tauschii*, *Brachypodium distachyon*, *A. thaliana*, *Cicer arietinum*, *Nicotiana sylvestris*, *Vitis vinifera*, and *Ziziphus jujuba*. TSV2 was very similar to many species and highly conserved in different higher plants (Figure 5A). Phylogenetic analysis showed that the evolutionary relationships of TSV2 homologs were consistent with the taxonomy (Figure 5B). Also, the mutated amino acid (Lys, K) in TSV2 was highly conserved among higher plants (Figure 5A) and its Lys-to-Arg mutation created the change of a β sheet structure (Supplementary Figure S2). This demonstrated the importance of this site for the functional integrity of the TSV2 protein.

### Expression pattern and subcellular localization of TSV2

**Figure 6:**
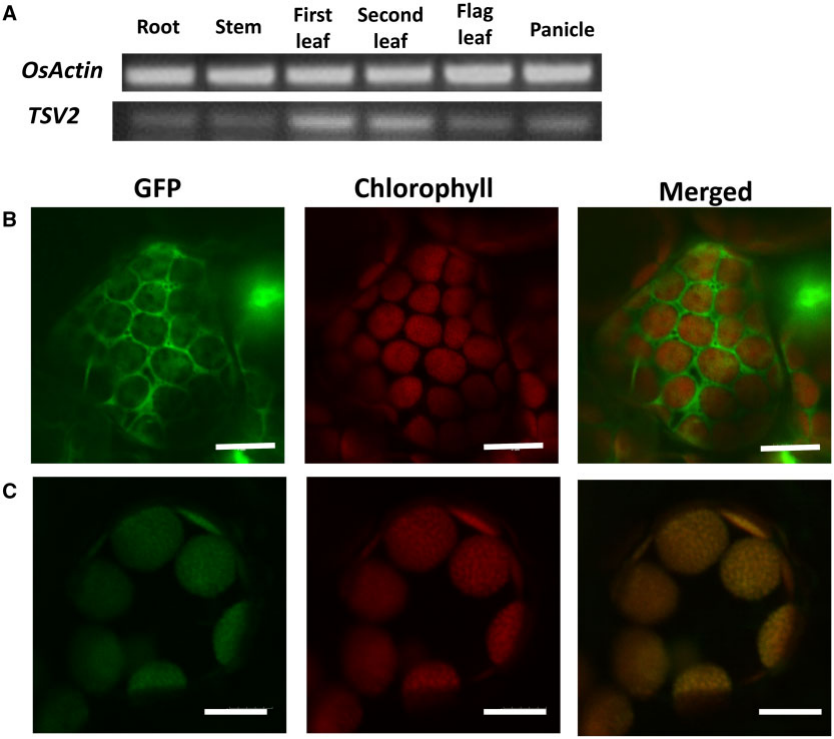
Expression pattern and subcellular localization of *TSV2.* (A) Analysis of expression of *TSV2* in different tissues by RT-PCR. *OsActin* was used as a control (the cycle number for *OsActin* was 28, the cycle number for *TSV2* was 35). (B) Empty GFP vector without a specific targeting sequence. (C) TSV2-GFP fusion. The scale bar represents 20 μm.

To clarify the expression pattern of *TSV2* in rice, a semi-quantitative RT-PCR was carried out with various tissues (Figure 6). Consistent with the rice gene expression profiling data in the RiceXPro database (Supplementary Figure S3), *TSV2* was highly expressed in the 1st and 2nd leaves, and weakly signaled in flag-leaf, root stem, and panicle (Figure 6A), showing the tissue-specific expression of *TSV2*. In addition, the TSV2 protein was predictively localized in the chloroplast using TargetP program (http://www.cbs.dtu.dk/services/TargetP/) (Emanuelsson *et al.* 2000). To verify the actual subcellular localization, the pMON530:CaMV35S:TSV2-GFP plasmid was introduced into tobacco cells in the transient expression assay, with the pMON530:CaMV35S-GFP vector as control. Observationally, the GFP fluorescence was co-localized with chlorophyll auto-fluorescence (Figure 6, B and C), affirming the localization of TSV2 in the chloroplast.

### Versatility for TSV2 function

**Figure 7:**
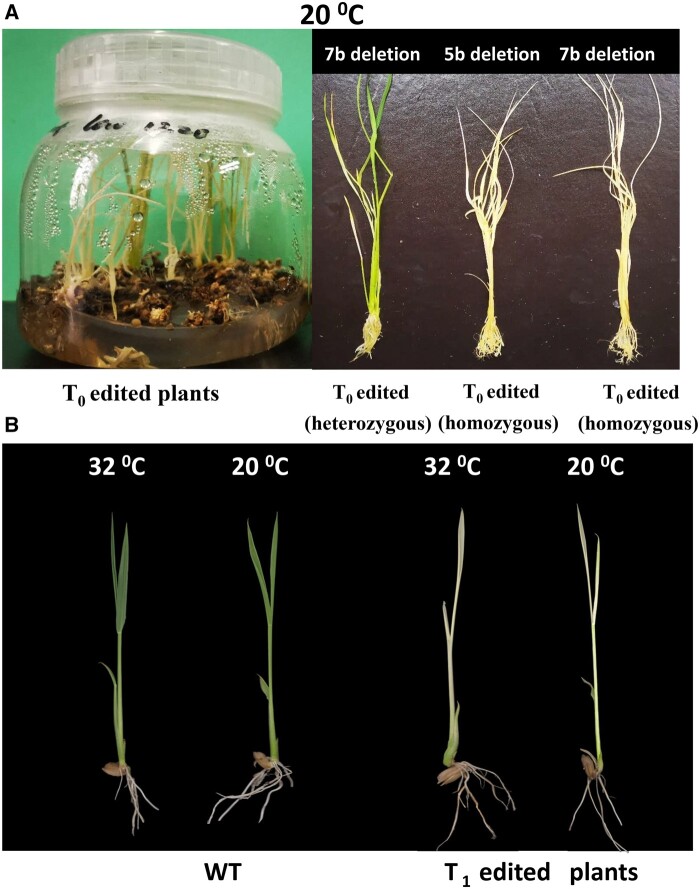
Phenotype of the edited transgenic plants by CRISPR/Cas9 technology. (A) T_o_ edited transgenic plants at 20°C. (B) The homozygous T_1_ plants from the heterozygous T_0_ plants grown at 20 and 32°C.

Zhang *et al.* (2017) early reported the existence of an allelic albino-lethal mutant (*las*) regardless of temperatures in rice, which is significantly different from the *tsv2* phenotype reported in this study. This might imply the versatility for the function of *LOC_Os02g33500(TSV2/LAS*), depending on where its mutation site located. We performed targeted mutation of *TSV2* gene in WT plants by CRISPR/Cas9 system. Surprisingly, all homozygous T_0_-edited transgenic seedlings, with two different edited-sites (5 and 7 base deletion, respectively) on the 1st exon of *TSV2* gene (Supplementary Figure S4), displayed albino phenotype and eventually died when grown at 20°C (Figure 7A). In addition, all T_1_-edited homozygous seedlings from T_0_-edited heterozygous transgenic seedlings displayed all albino phenotype and finally died when grown at 20 and 32°C (Figure 7B). Owing to the exon 1 sequence determined the ThrRS core domain (Figure 4C, Supplementary Figure S1); thus, the loss of function in the core domain of *LOC_Os02g33500* fatally led to albino death, regardless of temperatures. This is consistent with allelic *las* mutant (Zhang *et al.* 2017). Therefore, the expression of the core domain is co-regulated by other domains and temperatures, indicating the versatility for *TSV2* function for chloroplast development and seedling growth.

### The disruption of *TSV2* alters the transcript levels of associated genes

**Figure 8:**
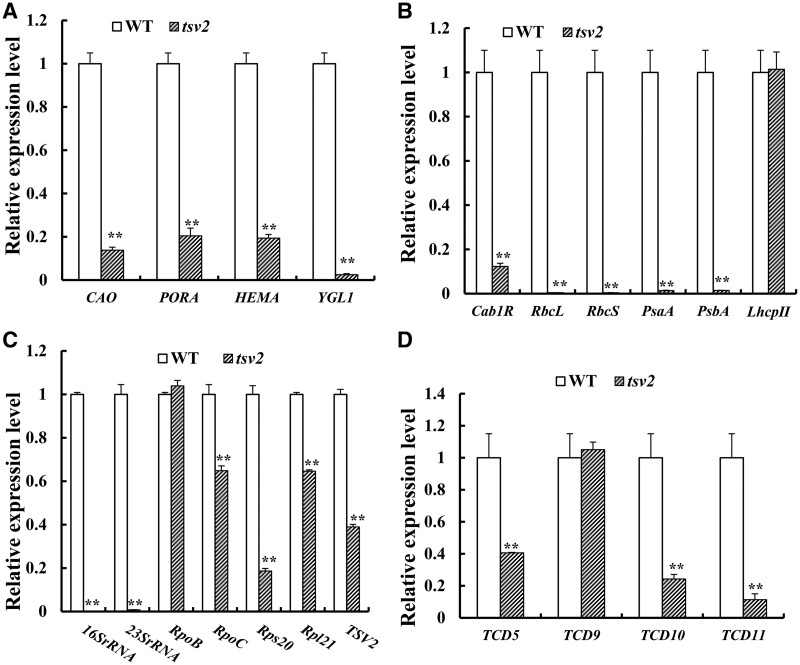
Quantitative expression analysis of the genes associated with Chl biosynthesis, photosynthesis, and chloroplast development in mutant at 20°C. (A–D) Expression levels of genes related to Chl biosynthesis, photosynthesis, chloroplast development and temperature sensitivity in WT and the *tsv2* mutant in the 3rd leaves, respectively. The relative expression level of each gene in WT and mutant was analyzed by qPCR and normalized using the *OsActin* as an internal control. Data are mean ± SD (*n* = 4). Asterisks indicate statistically significant difference compared with WT by Student’s *t*-test. *P <* *0.05 and **P <* *0.01 are indicated.

**Figure 9:**
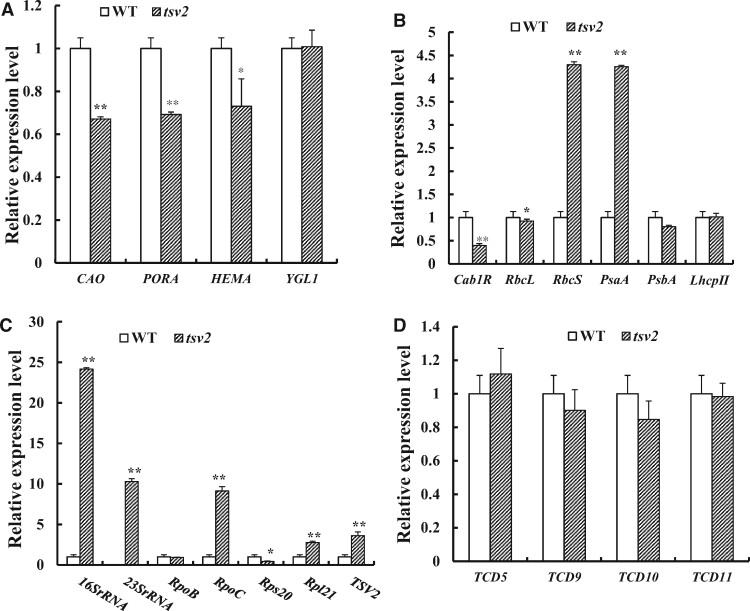
Quantitative expression analysis of the genes related to Chl biosynthesis, photosynthesis and chloroplast development in mutant at 32°C. (A–D) Expression levels of genes related to Chl biosynthesis, photosynthesis, chloroplast development and temperature sensitivity in WT and the *tsv2* mutant in the 3^rd^ leaves, respectively. The relative expression level of each gene in WT and mutant was analyzed by qPCR and normalized using the *OsActin* as an internal control. Data are mean ± SD (*n* = 4). Asterisks indicate statistically significant difference compared with WT by Student’s *t*-test. *P <* *0.05 and **P <* *0.01 are indicated.

To elucidate the effect of the *tsv2* mutation on the expression of genes related to chloroplast development and to explore the regulating pathway, we performed RT-qPCR analysis of 20 genes involved in chlorophyll biosynthesis, photosynthesis, chloroplast development, and temperature sensitivity in rice (Supplementary Table S2). It was observed that transcript levels of all tested genes for Chl biosynthesis, i.e., *chlorophyllide a oxygenase1* (*CAO1*), *glutamyl tRNA reductase* (*HEMA*), *NADPH-dependent protochlorophyllide oxidoreductase* (*PORA*), and *Chl synthetase* (*YGL1*), and for photosynthesis (*Cab1R*, *RbcL*, *PsaA*, *PsbA*, *LhcpII*) were significantly downregulated under low temperatures (20°C) (Figure 8, A and B), aligned with the observed albino phenotype of *tsv2* mutants (Figure 1); Also, at 20°C, the transcript levels of *16SrRNA* (small subunit) and *23SrRNA* (large subunit) involved in ribosome assembly and *Rps20*, encoding the small ribosomal subunit S20, in chloroplast, were greatly blocked (Figure 8C). Furthermore, at 20°C, except for *TCD9* encoding chloroplast chaperone protein OsCpn60α subunit (Jiang *et al.* 2014), three temperature-sensitive genes (*TCD5*, *TCD10*, and *TCD11*) for chloroplast development (Wang *et al.* 2016a, 2017; Wu *et al.* 2016) showed a significant downward trend of transcripts, in particular, *TCD10*, encoding PPR protein, and *TCD11*, encoding plastid ribosomal protein S6 (Figure 8D). Conversely, all transcripts of the reduced genes at 20°C in the *tsv2* seedlings recovered to or even exceeded WT levels (within twofold range) when grown at 32°C (Figure 9, A–D), coincident with the recovery of leaf-color (Figure 1D) and chloroplast development (Figure 3, G and H) in *tsv2* at 32°C. As such, our data revealed that the *tsv2* mutation leads to dramatic downregulation of many genes for chlorophyll biosynthesis, photosynthesis, and chloroplast development, under cold stress. In addition, the *TSV2* influences chloroplast ribosome assembly thereby affecting the process of chloroplast development and accumulation of photosynthetic pigment.

## Discussion

In this study, we identified and characterized rice *tsv2* mutants with imperfect chloroplasts and albino lethal phenotype at low temperatures, resulting from the abnormal expression of genes associated with chlorophyll biosynthesis, photosynthesis, and chloroplast development. The *tsv2* mutant phenotype was caused by the Lys-to-Arg mutation in anticodon binding domain in *TSV2* gene. However, the loss of function in core domain for TSV2 fatally led to seedling death regardless of temperatures. Our data evidenced that *TSV2* plays a critical role in chloroplast development and plant growth in rice.

### Incomplete of anticodon-binding domain in TSV2 leads to the albino phenotype under cold stress

To date, a lot of temperature-sensitive seedling-color mutants of rice similar to *tsv2* phenotype have been reported, with phenotypic white or yellow leaves at low temperatures while become normal green at high temperatures. Mutant *v1* and *v2* showed yellow leaf at 20°C and recovered to WT plants at 30°C (Iba *et al.* 1991; Kusumi *et al.* 1997). The coding protein of *V1* gene participates in the regulation of plastid RNA metabolism and protein translation (Kusumi *et al.* 1997). Guanylate kinase encoded by *V2* gene plays a special important role in the early development of chloroplasts (Sugimoto *et al.* 2007). Also, at 22°C, *osv5a* leaves yellowed and whitened, but turned to green phenotype over 28°C. *OsV5A* gene encodes chaperone protein interacting with PORA and PORB and stabilizing PORB protein (Zhou *et al.* 2013; Liu *et al.* 2016). Besides, the mutant *dua1* showed a pale phenotype at 19°C, PPR protein *DUA1* is essential for a chloroplast ribosome development under cold stress (Cui *et al.* 2019). Moreover, our research group also reported some thermo-sensitive leaf color mutants of rice. For example, *tcd9* mutant presented albino phenotype before the 3-leaf stage over 24°C and *TCD9* encodes chloroplast chaperone protein OsCpn60α subunit (Jiang *et al.* 2014). Mutants of *tcd3*, *tcd5*, *tcd10*, *tcd11*, *tcd33*, *tsv3*, and *osv4* exhibited albino or white phenotype and malformed chloroplast at 20°C, but return to normal phenotype at 32°C (Gong et al. 2014; Jiang et al. 2014; Wu et al. 2016; Wang et al. 2016a,b, 2007, 2020; Lin et al. 2018, 2020). More interestingly, *TCD5*, *TCD10*, and *OsV4* encode respective novel PPR protein (Gong *et al.* 2014; Wang *et al.* 2016b; Wu *et al.* 2016). Notwithstanding, different from the aforementioned mutants, the edited-mutant seedlings for *TSV2* gene in this study did not present temperature-sensitivity for leaf-color but died at high temperatures (Figure 7B). Thus, it is interesting to reasonably explore why allelic *las* mutant (Zhang *et al.* 2017) and the *TSV2*-edited mutants showed different phenotypes from the *tsv2* mutant. This is because that, whether edited-transgenic plants or *als* mutants, their mutation sites occur in the core ThrRS domain (Supplementary Figures S1 and S4). However, the mutation site of *tsv2* was located on the terminal exon 8, which only destroyed an original β sheet of the anticodon-binding domain in TSV2 (Supplementary Figure S2). Among AARSs, anticodon-binding domain is found in histidyl, glycyl, threonyl, and prolyl tRNA synthetases (Wolf *et al.* 1999) and involved in anticodon stem-loop binding and recognition (Aberg *et al.* 1997). In this study, the destruction of the core domain will lead to the full loss of the function of TSV2, as its mutant phenotype has nothing to do with temperatures. Interestingly, the Lys-to-Arg mutation in the anticodon-binding domain only resulted in whitening under cold stress and the increase of temperature can make up for the defect of its domain function. Therefore, the mutations of different domains in TSV2 will result in different phenotypes. Conclusively, the incomplete of anticodon-binding domain is responsible for the *tsv2* albino phenotype under cold stress.

### *TSV2* functions at the first step of chloroplast development

It is well known that chloroplast development is divided into three steps (Kusumi *et al.* 2010a,b; Kusumi and Iba, 2014). The first step involves the activation of plastid replication and plastid DNA synthesis. The second step involves the establishment of chloroplast genetic system. The last step involves the high expression of plastid and nuclear targets encoding the photosynthetic apparatus, resulting in the synthesis and assembly of the photosynthetic apparatus. At present, through utilization of thermo-sensitive leaf-color rice mutants, it was shown that *V3* (Yoo *et al.* 2009), *TCD9* (Jiang *et al.* 2014), *TCD10* (Wu *et al.* 2016), *TCD11* (Wang *et al.* 2017), *TSV3* (Lin *et al.* 2018a), *TCD33* (Wang *et a*l. 2020), and *TCD3* (Lin *et al.* 2020) function in the first step; *V1* (Kusumi *et al.* 1997), *V2* (Sugimoto *et al.* 2007), *TCD5* (Wang *et al.* 2016a), and *OsV4* (Gong *et al.* 2014) were involved in the second step; and *TCM12* (Lin *et al.* 2019) in the third step. Those findings showed that cold stress affects all three steps of chloroplast development, and its regulatory pathways are complex and diverse. To assess which step of chloroplast development was regulated by *TSV2*, the transcript levels of certain known genes for the first step (*TCD9*, *TCD10*, *TCD11*), the second step (*TCD5*), and the third step (*Cab1R*, *RbcL*, *PsaA*, *PsbA*, *LhcpII*) of chloroplast development in *tsv2* albino leaves under stress were observed in this study. Obviously, the transcript levels of the first step *TCD10* and *TCD11* genes were largely downregulated (Figure 8D), indicating that *TSV2* functions in the first step of chloroplast development. However, *TCD9*, which also involved in the first step, was still highly expressed (Jiang *et al.* 2014). This was probably attributed to that *TSV2* is localized between the downstream of *TCD9* and the upstream of *TCD10* and *TCD11* and was not regulated by *TCD9*. In addition, those inhibitions (*TCD10*, *TCD11*) caused by *TSV2* dysfunction in the first step, would definitely lead to the inhibition of certain associated-genes in its second (*TCD5*) and third (*Cab1R*, *RbcL*, *PsaA*, *PsbA*, *LhcpII*) stages (Figure 8, A and B), resulting in the reduction of chlorophyll accumulation and photosynthesis, consequently, seedling death.

### Possible role of TSV2 in chloroplast ribosome assembly and protein synthesis

Chloroplast development is a complex biological process, in which many key proteins are translated and formed in chloroplasts. The plastid ribosomal proteins are crucial to ribosome biosynthesis, plastid protein biosynthesis, chloroplast development, and ribosome assembly (Zhao *et al.* 2016). In the past, several genes for chloroplast ribosome assembly were reported in rice, such as *WLP1* encoding ribosome protein L13, RPL13 (Song *et al.* 2014), *ASL1* encoding ribosomal protein S20, RPS20 (Gong *et al.* 2013), *ASL2* encoding ribosomal protein L21, RPL21 (Lin *et al.* 2015), and *TCD11* encoding plastid ribosomal protein S6, RPS6 (Wang *et al.* 2017). As we have known, the mutation of *TCD11/Rps6* (Wang *et al.* 2017) and *WLP1/Rpl13* (Song *et al.* 2014) produced the thermo-sensitive leaf-color as the *tsv2* mutants, but the mutation of *ASL1/Rps20* (Gong *et al.* 2013) and *ASL2/Rpl21* (Lin *et al.* 2015) led to seedling death, regardless of temperatures, indicating that chloroplast ribosome subunits have different functions or responses to temperatures. In this study, the expression of *Rps20/ASL1*, *Rpl21/ASL2*, and *Rps6/TCD11* in *tsv2* mutants at low temperatures decreased greatly, evidently suggesting that *TSV2* affected chloroplast ribosome synthesis under cold stress. Notably, *16SrRNA* and *23SrRNA* were severely hampered (Figure 8C), definitely resulting in impaired translation and protein synthesis in chloroplasts. With the increase in temperature, all expressions of three ribosome subunit genes (*Rps20/ASL1*, *Rpl21/ASL2*, *Rps6/TCD11*) gradually returned to normal levels, especially for the expression of chloroplast ribosome *16SrRNA* and *23S rRNA* (Figure 9C). It is recognized that chloroplast translation occurs in 30S and 50S ribosomes; both are important components and their changes will directly affect ribosome assembly. Therefore, in the light of the reduced transcripts of *16SrRNA*, *23SrRNA*, *Rps20*, *Rpl21*, *TCD11/Rpl6* in albino *tsv2* seedlings (Figure 8, C and D), this once again proved that *TSV2/LAS* is involved in the chloroplast ribosomes assembly and protein synthesis, consistent with Zhang *et al.* (2017). In conclusion, our data clearly revealed that the *TSV2* encodes a rice ThrRS, which is located in chloroplasts and is closely related to the assembly of chloroplast ribosomes and functions at the first step of chloroplast development. Mutation in the anticodon-binding domain of TSV2 causes chloroplast ribosomes to fail to assemble normally, resulting in impaired chloroplast development and lethal phenotype of rice seedlings under cold stress, but the loss of function in the core domain fatally led to seedling death, regardless of temperatures. Further work is warranted to explore from mechanism the roles of *TSV2* in chloroplast development and plant growth.

## Supplementary Material

jkab196_Supplementary_Data
