# Supplementary material for: Rice TSV2 encoding threonyl-tRNA synthetase is needed for early chloroplast development and seedling growth under cold stress
Source: G3 (Bethesda). 2021 Jul 30;11(9):jkab196. doi: 10.1093/g3journal/jkab196 (PMC8661440; doi:10.1093/g3journal/jkab196)
Supplement: jkab196_Supplementary_Data [file jkab196_supplementary_data.zip › jkab196-suppl_data/GENETICS-G3-2020-402084-s08.docx]

## **Supplementary information**

**Table S1** The PCR-based molecular markers designed for mapping.

**Table S2** Markers designed for real-time qPCR and gene functions.

**Table S3** Genetic segregation analysis of *tsv2* mutants in the F_2_ population

**Figure S1** The domains of *TSV2/LAS* and mutation and edited sites

**Figure S2** Prediction of protein spatial structure of TSV2 in WT and *tsv2* mutants (http://www.sbg.bio.ic.ac.uk/phyre2/html/page.cgi?id=index)

**Figure S3** Expression patterns of *TSV2*(*LOC_Os02g33500*). Data were cited from the rice expression profile database, RiceXPro (http://ricexpro.dna.affrc.go.jp/category-select.php).

**Figure S4** Full-length cDNA sequence of LOC_Os02g33500 (*TSV2/LAS*) and mutation sites
