# Supplementary material for: Rice TSV2 encoding threonyl-tRNA synthetase is needed for early chloroplast development and seedling growth under cold stress
Source: G3 (Bethesda). 2021 Jul 30;11(9):jkab196. doi: 10.1093/g3journal/jkab196 (PMC8661440; doi:10.1093/g3journal/jkab196)
Supplement: jkab196_Supplementary_Data [file jkab196_supplementary_data.zip › jkab196-suppl_data/GENETICS-G3-2020-402084-s01.doc]

**Figure S1.** The domains of TSV2/LAS and positions of mutated and edited sites

MAAAVSAPLLRFHHHHRCLLRHSSSPRRYPAGFSRGLSSVSFGLPRSTTTAARSTASPSA

PSAAAAEATDAASAQAGSD**G**KGDGIGEEERVVLPTNESSDRLLRIRHTCAHVMAMAVQKL

FPNSKVTIGPWIDNGFYYDFDMEPLTDKDLKRIKKEMDRIIRKNLPLVREEVSREEAQKR

IEALNEPYKLEILEGIKEDPITIYHIGEEWWDLCAGPHVESTGKIDRRAVELESVAGAYW

RGDESNQMLQRIYGTAWETEDQLKAYIHFKEEAKRRDHRRLGLDLDLFSI**QE**DAGGGLVF

WHPKGAIIRHVLEDSWKQIHLQRGYDLLYTPHVAKAELWKVSGHIDFYKENMYNQMDVED

ELYQLRPMNCPYHILVYKRKLHSYRDFPIRVAELGTVYRYELSGSLHGLFRVRGFTQDDA

HIFCLEDQIKDEIRGVLDLTEQILGQFGFRYYEINLSTRPEKSVGSDDIWEKATLALKDA

LEDKGWEYKVDEGGGAFYGPKIDLKIEDALGRKWQCSTVQVDFNLPERFDITYVNSNSEK

KRPIMIHRAILGSLERFFGVLIEHYAGDFPLWLAPIQARILPVTDNELQYCNEVASELKS

KGIRAEVCHGERLP**K**LIRNAETKKVPLMAVVGPKEVQARTLTIRSRHSGEIGTMPVDEFF

RRIQLAIANKSSSL

**Note:** The black and red underlines indicated the location of threonine tRNA synthetase (ThrRS) domain and anticodon binding domain, respectively. The **red aa (G) site** barely represents the start of the edited position in this study, and the **blue aa (QE)** site represents the mutation location in multiple allelic *las* mutant (Zhang et al. 2017), both which led to the loss of the function of ThrRS domain; **the green aa (K) site** indicates the Lys(K)-to-Arg(R) mutation site in *tsv2* mutants in this study.
