# Supplementary material for: Rice TSV2 encoding threonyl-tRNA synthetase is needed for early chloroplast development and seedling growth under cold stress
Source: G3 (Bethesda). 2021 Jul 30;11(9):jkab196. doi: 10.1093/g3journal/jkab196 (PMC8661440; doi:10.1093/g3journal/jkab196)
Supplement: jkab196_Supplementary_Data [file jkab196_supplementary_data.zip › jkab196-suppl_data/GENETICS-G3-2020-402084-s02.doc]

**Figure S2.** Prediction of protein spatial structure of TSV2 in WT and *tsv2* mutant

**WT	*tsv2* mutant**
