# Supplementary material for: Rice TSV2 encoding threonyl-tRNA synthetase is needed for early chloroplast development and seedling growth under cold stress
Source: G3 (Bethesda). 2021 Jul 30;11(9):jkab196. doi: 10.1093/g3journal/jkab196 (PMC8661440; doi:10.1093/g3journal/jkab196)
Supplement: jkab196_Supplementary_Data [file jkab196_supplementary_data.zip › jkab196-suppl_data/GENETICS-G3-2020-402084-s04.doc]

**Figure S4.** Full-length cDNA sequence of LOC_Os02g33500 (*TSV2/LAS*) and mutation sites

ATGGCGGCCGCCGTCTCCGCGCCCCTTCTCCGGTTCCACCACCACCACCGCTGCCTCCTC

CGCCACTCCTCCTCGCCGCGCCGCTACCCAGCCGGCTTCTCGCGTGGGCTCAGCTCCGTC

TCGTTCGGCCTCCCGCGCAGCACCACCACCGCCGCGCGCTCCACCGCCTCGCCCTCGGCG

CCGTCGGCGGCGGCGGCCGAGGCCACGGATGCGGCTTCCGCCCAGGCGGGATCCGACGG**C**

**AAGG**GGGATGGGATTGGGGAGGAGGAGCGCGTCGTGCTCCCCACCAACGAGTCCTCCGAC

CGCCTGCTCCGCATCCGCCACACCTGTGCGCATGTGATGGCCATGGCCGTCCAGAAGCTG

TTCCCCAACTCCAAGGTGACAATAGGTCCCTGGATAGACAACGGCTTCTACTACGACTTC

GACATGGAGCCCCTCACGGACAAGGACCTCAAGAGGATCAAGAAAGAGATGGATCGGATC

ATCCGGAAGAATCTCCCATTGGTGAGGGAGGAGGTGTCCAGGGAAGAGGCCCAAAAGAGG

ATTGAAGCGCTTAATGAGCCATATAAGTTGGAAATTTTGGAGGGCATCAAGGAAGATCCT

ATCACAATCTATCACATTGGAGAAGAGTGGTGGGATCTCTGTGCCGGACCTCATGTCGAG

TCTACTGGCAAAATCGATAGAAGAGCTGTTGAGCTTGAGTCTGTTGCTGGTGCTTACTGG

AGAGGTGACGAAAGTAATCAAATGCTACAAAGAATATACGGGACTGCATGGGAGACTGAA

GACCAATTGAAGGCTTACATCCACTTCAAGGAAGAGGCCAAGCGCCGTGATCATCGGCGA

TTAGGTCTGGATCTTGATCTATTCTCCATACAGGAAGATGCTGGTGGCGGTTTAGTATTC

TGGCATCCAAAAGGTGCTATTATCAGACATGTTTTAGAAGACTCATGGAAACAGATTCAC

TTGCAACGTGGTTATGATCTATTATACACTCCCCACGTTGCAAAGGCTGAACTTTGGAAG

GTTAGTGGGCATATCGATTTCTACAAAGAGAACATGTACAATCAAATGGATGTTGAAGAT

GAGCTATATCAACTTCGGCCCATGAATTGCCCTTACCACATTTTAGTATACAAGAGAAAG

CTACATTCATACAGGGATTTTCCCATTCGAGTGGCAGAGCTTGGAACTGTCTACAGATAT

GAGCTATCTGGTAGTTTGCA**TGGGC**TGTTCCGTGTCAGAGGATTTACGCAGGATGATGCC

CACATATTTTGTCTGGAGGACCAGATAAAAGATGAAATTAGAGGTGTTCTTGATCTGACT

GAGCAAATACTGGGCCAATTTGGCTTTCGGTATTATGAGATAAACCTTTCAACAAGGCCA

GAAAAATCTGTTGGCAGTGATGACATATGGGAAAAAGCAACACTTGCACTAAAAGATGCA

CTAGAAGACAAAGGTTGGGAGTACAAGGTTGATGAAGGTGGAGGCGCTTTCTATGGCCCA

AAAATTGATTTGAAAATTGAGGATGCTCTTGGAAGAAAATGGCAATGTTCCACTGTGCAG

GTCGATTTCAATTTACCTGAGCGGTTTGACATTACTTATGTCAATTCAAATAGCGAGAAG

AAACGGCCTATCATGATTCATAGAGCTATCCTTGGGTCTTTGGAACGATTTTTTGGTGTC

CTCATTGAACACTATGCTGGTGATTTTCCACTTTGGCTTGCACCAATCCAAGCTCGTATT

CTACCTGTGACAGACAATGAGCTGCAATACTGTAACGAGGTGGCTTCAGAACTGAAATCA

AAAGGCATTCGAGCTGAGGTATGTCATGGCGAGCGTCTACCA**A**AGCTAATACGGAATGCC

GAAACGAAGAAAGTGCCGCTCATGGCGGTCGTTGGGCCTAAAGAAGTTCAAGCCAGGACC

CTCACTATCAGGTCCAGGCATAGTGGGGAGATTGGTACTATGCCTGTGGATGAATTCTTT

CGCAGAATCCAACTTGCTATTGCGAACAAATCTTCTTCACTATGA

**Note:** The underline 7b sequence (GGCAAGG) and red 5b sequence (CAAGG) indicated the deletion sites in edited T_0_ plants caused by CRISPR/Cas9 system, respectively; the double underline sequences for **TGGG** and A indicate the mutation site in *las* (Zhang *et al*. 2017) and *tsv2* mutants, respectively.
