# Supplementary material for: Rice TSV2 encoding threonyl-tRNA synthetase is needed for early chloroplast development and seedling growth under cold stress
Source: G3 (Bethesda). 2021 Jul 30;11(9):jkab196. doi: 10.1093/g3journal/jkab196 (PMC8661440; doi:10.1093/g3journal/jkab196)
Supplement: jkab196_Supplementary_Data [file jkab196_supplementary_data.zip › jkab196-suppl_data/GENETICS-G3-2020-402084-s05.doc]

**Table S1.** The PCR-based molecular markers designed for mapping

| Marker | Name | Primer sequences (5’-3’) |
| --- | --- | --- |
| SSR | MM2855 | GCCGCCTTCTTCTTTTTCTC |
|  |  | GATTCCACGAGAAGGTTTGC |
|  | MM3223 | GAATGGACGACATGTACGACACC |
|  |  | GGATGACGGACGAAAGCTAAGG |
|  | MM3274 | AATGGGATATAGAGGCTGTTGC |
|  |  | TACTAGCAGTCTTCTCACCTGAAGC |
|  | MM3298 | ATCAAGCAAACCAAGGCATC |
|  |  | TTTGCTTAGCTTGGCCTCTC |
|  | MM3457 | ATGCAATCGGTGCTGACATA |
|  |  | TAACCACGTCGATGCCAATA |
|  | MM3897 | GCGCACTCCGATAGGAATTA |
|  |  | GTCAAGAACAGCAACGCAAG |
| Indel | ID12613 | TGCTTAACTTCTCCAACACC |
|  |  | TCTACATGTCTTCATTACCA |
|  | ID12947 | CTCTAGCGACACACACGATA |
|  |  | CAATACAAATGCAACCTAGC |
|  | ID13097 | ACCCTGTATATGGTTGGTGA |
|  |  | CCCATGTTACAGTACGAGAC |
|  | ID19911 | AAACTACAGGGTCCAATTTT |
|  |  | CGTTTTTGCCATTGTACTTT |
