# Supplementary material for: Rice TSV2 encoding threonyl-tRNA synthetase is needed for early chloroplast development and seedling growth under cold stress
Source: G3 (Bethesda). 2021 Jul 30;11(9):jkab196. doi: 10.1093/g3journal/jkab196 (PMC8661440; doi:10.1093/g3journal/jkab196)
Supplement: jkab196_Supplementary_Data [file jkab196_supplementary_data.zip › jkab196-suppl_data/GENETICS-G3-2020-402084-s06.doc]

**Table S2.** Markers designed for real-time qPCR and gene functions.

| Gene | Primer sequences (5’-3’) | Gene function |
| --- | --- | --- |
| ***TSV2*** | GACCAATTGAAGGCTTACATCC | Encoding a threonyl-tRNA synthetase in this study |
|  | CAGCATCTTCCTGTATGGAGAA |  |
| ***Cab1R*** | AGATGGGTTTAGTGCGACGAG | Encoding light-harvesting chlorophyll alb binding protein 1 |
|  | TTTGGGATCGAGGGAGTATTT |  |
| ***CAO*** | GATCCATACCCGATCGACAT | The chlorophyllide a oxygenase in Chl b biosynthesis |
|  | CGAGAGACATCCGGTAGAGC |  |
| ***LhcpII*** | GAAGAAGATCAAGAACGGCC | Encoding light harvesting chlorophyll a⁄b binding protein of PSII |
|  | TTGCCGGGGACGAAGTTGGT |  |
| ***PORA*** | TGTACTGGAGCTGGAACAACA | Encoding pchlide oxidoreductase (POR)  (Sperling, et al.1997) |
|  | GAGCACAGCAAAATCCTAGACG |  |
| ***PsaA*** | GCGAGCAAATAAAACACCTTC | Encoding the P700 apoproteins of photosystem I (Steiner et al. 2009) |
|  | GTACCAGCTTAACGTGGGGAG |  |
| ***PsbA*** | CCCTCATTAGCAGATTCGTTTT | Encoding the D1 protein of photosystem II (Steiner et al. 2009) |
|  | ATGATTGTATTCCAGGCAGAGC |  |
| ***RbcL*** | CTTGGCAGCATTCCGAGTAA | Encoding the large subunit of Rubisco  (Barkan et al.1993) |
|  | ACAACGGGCTCGATGTGATA |  |
| ***Rpl21*** | AAGAAGAGGAGGCTGCGGT | Encoding the plastid ribosomal protein L21  ( Lagrange et al.1997; Lin et al., 2015) |
|  | GACATTGGCGCCTTTCAGC |  |
| ***RpoB*** | TTTGGTTTCGATGTGCA | Encoding RNA polymerase β subunits of PEP  (Little et al. 1988) |
|  | TATGGTCTAATTCCGAGCGGT |  |
| ***RpoC*** | ATTAGACGCATGCAATTGGC | Encoding RNA polymerase β' subunits of PEP  (Little et al. 1988) |
|  | CAATGGGTCTTAATTCGGGA |  |
| ***Rps20*** | CACGCTCTTCTCCCTCTCCT | Encoding a component (S20) of the small ribosomal subunit |
|  | GTAGGAGGCGGACAGGCG |  |
| ***TCD5*** | CTCCCAACCAGACACTATCA | Encoding a monooxygenase, affects chloroplast development at low temperatures(Wang et al., 2016a) |
|  | CGCCCTTTATGTACTTCAGC |  |
| ***TCD9*** | ATGACTCTGAAGAGAAGATTGGTGT | Encoding α subunit of chaperonin protein 60 (Cpn60α) for the chloroplast development(Jiang et al. 2014) |
|  | ATTGCATTGTATCCAACTCGCCATT |  |
| ***TCD10*** | TCGGTTGTGGTCCAGATGAGT | Encoding a novel PPR protein(Wu et al. 2016 ) |
|  | ATCTGGGTTTATTCCCTGGTC |  |
| ***TCD11*** | GCGAGCTCAACAACAGTATATG | Encoding plastid ribosomal protein S6 is essential for chloroplast development(Wang et al. 2017) |
|  | TACCTCAAGACTTTCTCAGCTG |  |
| ***YGL1*** | CAGTCTCCAATGGCCACCT | Encoding chlorophyll synthesis(Wu et al.2007) |
|  | TGCTTTCATCAGTGGCTGGT |  |
| ***16sRNA*** | CCGTTGGTGTTCTTTCCGAT | Encoding chloroplast ribosomes small subunits of 16S components |
|  | TTCAAGTCCGCCGTCAAATC |  |
| ***23sRNA*** | TGTGGGCGTTAGAGCATTGAG | Encoding chloroplast ribosomes large subunits of 23S components |
|  | CACTTGGCTACCCAGCGTTTA |  |
| ***Actin*** | AGGAAGGCTGGAAGAGGACC | Encoding chlorophyll synthesis (Wu et al.2007) |
|  | CGGGAAATTGTGAGGGACAT |  |
