# Supplementary material for: Rice TSV2 encoding threonyl-tRNA synthetase is needed for early chloroplast development and seedling growth under cold stress
Source: G3 (Bethesda). 2021 Jul 30;11(9):jkab196. doi: 10.1093/g3journal/jkab196 (PMC8661440; doi:10.1093/g3journal/jkab196)
Supplement: jkab196_Supplementary_Data [file jkab196_supplementary_data.zip › jkab196-suppl_data/GENETICS-G3-2020-402084-s07.doc]

**Table S3**. Genetic segregation analysis of *tsv2* mutants in the F_2_ population.

| Cross | Observed number of F_2_ plants | | | χ^2^_(3:1)_ | *P* |
| --- | --- | --- | --- | --- | --- |
|  | Total | Green | Albino |  |  |
| Peiai64S/*tsv2* | 301 | 231 | 70 | 0.488 | *P*＞0.05 |

χ^2^_0.05_=3.84
